# Are payers treating orphan drugs differently?

**DOI:** 10.3402/jmahp.v2.23513

**Published:** 2014-01-15

**Authors:** Joshua P. Cohen, Abigail Felix

**Affiliations:** Tufts Center for the Study of Drug Development, Boston, MA, USA

**Keywords:** orphan drugs, reimbursement, pricing

## Abstract

**Background:**

Some orphan drugs can cost hundreds of thousands of dollars annually per patient. As a result, payer sensitivity to the cost of orphan drugs is rising, particularly in light of increased numbers of new launches in recent years. In this article, we examine payer coverage in the United States, England and Wales, and the Netherlands of outpatient orphan drugs approved between 1983 and 2012, as well as the 11 most expensive orphan drugs.

**Methods:**

We collected data from drug regulatory agencies as well as payers and drug evaluation authorities.

**Results:**

We found that orphan drugs have more coverage restrictions than non-orphan drugs in all three jurisdictions. From an economic perspective, the fact that a drug is an orphan product or has a high per-unit price *per se* should not imply a special kind of evaluation by payers, or necessarily the imposition of more coverage restrictions.

**Conclusion:**

Payers should consider the same set of decision criteria that they do with respect to non-orphan drugs: disease severity, availability of treatment alternatives, level of unmet medical need, and cost-effectiveness, criteria that justifiably may be taken into account and traded off against one another in prescribing and reimbursement decisions for orphan drugs.

Historically, the high development costs associated with bringing a drug to market and limited potential for revenues discouraged companies from developing products for diseases impacting small numbers of patients. Rare disease populations were therefore ‘orphaned’, or neglected, and given relatively few treatment options. To address this public health need, in 1983 the US government passed the Orphan Drug Act (ODA) (1). With the establishment of 7 years of market exclusivity, development tax credits, and a shorter US Food and Drug Administration (FDA) approval period, the ODA lowers barriers to innovation and fosters development of treatments for diseases affecting fewer than 200,000 individuals. As such, the law has been an unequivocal success. To illustrate, in the decade prior to 1983 only 34 orphan products were marketed, whereas between 1983 and 2009 the FDA approved 275 orphan drugs for 337 orphan indications (2). In fact, during the 2000s, orphan products comprised 22% of all new molecular entities and 26% of all biologics receiving FDA approval (2, 3). In 2000, the European Medicines Agency (EMA) enacted similar orphan drug regulations, with a 10-year period of marketing exclusivity. In turn, this has led to a significant proliferation of orphan product approvals in Europe as well (4).

Some orphan drugs can cost payers hundreds of thousands of dollars annually per patient. Generally, with few alternative therapies available, payers have had reduced negotiating power *vis-à-vis* pricing (5, 6). Payer sensitivity to the cost of orphan drugs is rising, particularly in light of recent launches of high-cost products (7). As a result, orphan drugs are increasingly subject to formulary restrictions. For example, in recent years US payers have shifted from fixed co-payments per prescription to ‘co-insurance’ for most orphan drugs. That is, the patient's out-of-pocket expenses are calculated as a percentage of the drug's cost. This shift was designed to control prescription utilization and spending (8). Co-insurance percentages have on average risen from 15 to 28% in the past decade (9, 10). Given the level of prices of many orphan drugs, this represents a significant shift of the cost burden onto the patient. Also, in therapeutic classes with multiple orphan options, such as colorectal cancer and chronic myeloid leukemia, US payers are differentiating among orphan drugs by granting preferred formulary status to certain drugs and non-preferred status to others (10). Furthermore, payers are adding prior authorization to many orphan drugs as a condition of reimbursement, or requiring clinical diagnostic tests prior to prescribing. In Europe, orphan drug prices are under substantial pressure from health authorities. To illustrate, in the Netherlands, the Ministry of Health is asking drug manufacturers to voluntarily reduce prices to ‘acceptable’ price levels (11, 12). Moreover, in the United Kingdom, the National Institute for Health and Clinical Excellence (NICE) is reviewing the ‘very high price of drugs for people who suffer from rare conditions’ in an apparent attempt to force manufacturers to agree to price reductions when drugs do not perform as predicted (12). Orphan drug reimbursement is facing similar challenges, leading to increased numbers of coverage denials by other European health authorities, as well as other restrictions on reimbursement (13).

## Orphan drug coverage in the United States, England and Wales, and the Netherlands

To examine the current status of orphan drug coverage, we analyzed regulatory approval and reimbursement data concerning two subsets of orphan drugs: 1) outpatient orphan drugs approved between 1983 and 2012, and 2) orphan drugs costing payers over $225,000 annually per patient.

### Coverage of outpatient orphan drugs approved between 1983 and 2012

In the United States, Faden and Huskamp analyzed Medicare Part D (self-administered drugs) coverage of 99 outpatient orphan drugs approved by the FDA between 1983 and 2008 (14). They found a coverage rate of 85% of Medicare Part D payers. This implies that 15% of the drugs were not covered by at least one plan. This coverage percentage is lower than the average percentage of non-orphan drugs covered (15–17). Faden and Huskamp found that orphan drugs were usually placed in the highest cost-sharing tier, and almost invariably were subject to co-insurance in which patients pay a variable percentage of the cost of a medication instead of fixed co-payments per prescription. Most payers institute a maximum amount for which patients are responsible in terms of out-of-pocket costs. This can, however, be as much as tens of thousands of dollars per year per patient. Prior authorization was the most widely used form of utilization management. This condition of reimbursement requires that a prescriber obtain approval from a payer to prescribe a specific drug. Prior authorization was used by at least one plan for 80% of the covered orphan drugs.

Using Medicare's Formulary Plan Finder (18), we examined coverage by 10 leading Medicare Part D plans (the largest plans in terms of numbers of covered Medicare beneficiaries) of 17 outpatient orphan drugs approved from 2009 through 2012. We found very similar numbers. Three of 17 (18%) were not covered by at least one plan. Prior authorization was used by at least one plan for 82% of the 2009–2012 approvals.

Analyzing the set of 116 FDA-approved outpatient orphan drugs between 1983 and 2012, we found that in England and Wales, 98 drugs were approved by the British Medicines and Healthcare Products Regulatory Agency (MHRA) or EMA. Thirty-six orphan products were reviewed by NICE. Twenty-one were given positive recommendations, 15 negative recommendations (not reimbursed),and 12 conditionally reimbursed. The 42% rejection rate – 15 out of 36 – is higher than the coverage rate reported for non-orphan drugs in previous studies (15, 16). And the percentage of covered drugs that are conditionally reimbursed (33%) is higher as well. Official policy states that drugs approved by MHRA and not reviewed by NICE are by default covered by the National Health Service. However, this is evidently a flawed presumption in some instances. Patients may be denied access to certain orphan drugs that have been granted marketing authorization because NICE has not reviewed them (18). The majority of patients in England and Wales are exempt from any co-payment, while those who were not exempt pay a nominal fee of about £10 per prescription.

Examining the set of FDA-approved outpatient orphan drugs between 1983 and 2012, we found that in the Netherlands, 98 of the 116 drugs were approved by the Dutch Medicines Evaluation Board (CBG) or the EMA. Ninety-two of the 98 were reviewed by the Dutch Health Insurance Board (CVZ). Seventy-nine were covered, 13 (14%) were denied reimbursement, and 22 (24%) were conditionally reimbursed. The rejection rate of 14% and the percentage of conditionally reimbursed products (24%) are higher than the respective rates for non-orphan drugs (15, 16). Six drugs were approved and not reviewed. Formally, drugs approved by College ter Beoordeling Geneesmiddelen (CBG) and not reviewed by CVZ are by default covered in the Netherlands. Patients in the Netherlands were exempt from any co-payment for any of the covered drugs (Table I).

**Table I:** Orphan drug reimbursement in England and Wales and the Netherlands

	England and Wales	The Netherlands
Health authority	National Institute for Health and Clinical Excellence	Dutch Health Insurance Board
Number of drugs approved by drug regulatory agency (MHRA or CBG)	98	98
Number of drugs reviewed by health authority	36	92
Number of drugs covered*	21	79
Number of drugs rejected by health authority or by at least one plan	15	13
Number of drugs conditionally covered	12**	22

* Either covered by the Dutch Health Insurance Board or given a ‘positive recommendation’ by NICE.

** Six of the drugs were given indications restrictions; six were subject to so-called patient access schemes. Here, access is granted while clinical effectiveness and cost-effectiveness evidence are generated in a post-marketing study.

Sources: National Institute for Health and Clinical Excellence, http://nice.org.uk; Dutch Health Insurance Board, http://medicijnkosten.nl; Dutch Health Care Authority, http://nza.nl; Dutch Health Insurance Board, http://www.fk.cvz.nl; British Medicines and Healthcare Products Regulatory Agency, http://www.mhra.gov.uk/index.htm#page=DynamicListMedicines; Dutch Medicines Evaluation Board, http://www.cbg-meb.nl/cbg/en/default.htm.

### The over $225,000 club

Table II shows 11 orphan drugs that cost payers over $225,000 per US patient annually. All 11 are FDA approved. Nine are physician-administered injectables. Two are outpatient oral medications. Note that rilonacept, after being approved by the EMA, was withdrawn in Europe. We observe that in the United States, Medicare Part B covers the nine physician-administered drugs, while all Part D plans covered the two outpatient drugs. For Medicare Part B drugs, Medicare beneficiaries pay 20% co-insurance.^1^ On average, for the two Medicare Part D drugs, co-insurance was 28% across the 10 leading Part D plans. The Dutch CVZ placed eculizumab, imiglucerase, galsulfase, alglucosidase, idursulfase, agalsidase beta, and ivacaftor on its ‘special list’ of ‘expensive’ medications, with an earmarked budget. This follows a trend in Europe. Under pressure from patient advocacy groups and healthcare providers, to counteract the possibility of non-coverage of certain orphan drugs, a number of health authorities have allocated earmarked resources to the pharmaceutical treatment of certain rare diseases to ensure patient access (19). The C1 esterase inhibitor and rilonacept were not on the ‘special list’, the latter drug because it has been withdrawn from the market. Teduglutide is not reimbursed. And in late 2012, CVZ advised the Ministry of Health to exclude agalsidase beta from coverage for all indications and alglucosidase alfa from coverage for those suffering from the non-classic form of Pompe disease.^2^ In England and Wales, the C1 esterase inhibitor was approved by MHRA, but placed on the ‘Black Triangle’ list for ‘intense’ post-marketing surveillance. NICE did not review any of the 11 drugs listed in Table II. However, the British Minister of Health advised against coverage of eculizumab, despite no NICE assessment. As was said above, patients may be denied access to certain orphan drugs that have been granted marketing authorization in the absence of NICE reviews (20).

**Table II:** Eleven orphan drugs with annual costs per US patient of more than $225,000

Orphan drug (trade name)	Indication	Annual cost per patient, US$
Agalsidase beta (Fabrazyme)	Fabry disease	239,000
Lomitapide (Juxtapid)	Homozygous familial hypercholesterolemia	250,000
Rilonacept (Arcalyst)	Cryopyrin-associated periodic syndromes	250,000
Teduglutide (Gattex)	Short bowel syndrome	295,000
Imiglucerase (Cerezyme)	Type 1 Gaucher disease	300,000
Ivacaftor (Kalydeco)	Cystic fibrosis	325,000
Galsulfase (Naglazyme)	Mucopolysaccharidosis VI	441,000
Idursulfase (Elaprase)	Mucopolysaccharidosis I and II	475,000
Eculizumab (Soliris)	Paroxysmal nocturnal hemoglobinuria	486,000
C1 esterase inhibitor (Cinryze)	Hereditary angioedema prophylaxis	487,000
Alglucosidase alfa (Myozyme)	Pompe disease	575,000

Compiled from Herper (21), Hyde et al. (7), and Tilles et al. (22).

This study's most important limitation is that we included only two European countries in our analysis. Therefore, we cannot necessarily generalize from findings in the United Kingdom and the Netherlands to other European countries.

## Policy implications

From our analysis, we see that orphan drugs currently have more coverage restrictions than non-orphan drugs in the United States, United Kingdom, and Netherlands. Whether this can be considered a rational response to the rise in numbers of launches of costly orphan drugs remains to be seen. Health authorities may be responding to price independent of other factors, such as budget impact. Fears that growth in orphan drug expenditures will be unsustainable in the long run may not be justified, in part due to their limited budget impact (23). To illustrate, the products listed in Table II are very expensive in terms of per-unit price. Nevertheless, the total number of patients prescribed these drugs is quite low. In some cases, only a few hundred people in the United States and Europe are affected by the disease in question (e.g., mucopolysaccharidosis I, III, and VI). In others, such as Fabry disease, homozygous familial hypercholesterolemia, and type 1 Gaucher disease, only a few thousand individuals in the United States and Europe are impacted. Indeed, the share of the total pharmaceutical market represented by orphan drugs is presently quite low at 3.5% (23). It will likely reach 4.5% by 2016 (23). This percentage is expected to subsequently level off owing to a diminished growth rate in orphan drug approvals as well as patent expirations of existing orphan drugs.

From an economic perspective, therefore, it would not make sense to single out orphan drugs based on their per-unit price. Nor would it be sound policy to segment budgets in such a way that certain orphan drugs arbitrarily receive a special allotment. This kind of preferential treatment may benefit the loudest voices among patient advocates, which is not necessarily equitable. Surely, orphan drugs should be subject to similar types of clinical effectiveness, cost-effectiveness, and budget impact analyses as non-orphan drugs receive. Also, health authorities should provide justification of orphan drug reimbursement decisions using a myriad of criteria, some of which may be traded off against one another. In some instances, for example, budget impact may trump poor cost-effectiveness. In others, disease severity or lack of treatment alternatives may override poor cost-effectiveness. Hence, from a payer perspective, high-cost orphan drugs with treatment alternatives, such as the C1 esterase inhibitor, should be handled differently from, say, agalsidase beta, which has no such alternative. The same reasoning should apply to non-orphan drugs. In sum, the fact that a drug is an orphan product or has a high per-unit price *per se* should not imply a special kind of evaluation by payers (12).

Finally, there is a constructive model for improving patient access to orphan drugs using a forward-thinking, evidence-based approach: use of coverage with evidence development in cases in which evidence at launch is lacking, inconclusive, or controversial. Here, payers temporarily provide access to a product while reviewing its effectiveness and safety. They may revisit their decision to reimburse in light of newly generated evidence.

## Footnotes

1 Note that between 85 and 90% of Medicare beneficiaries have supplemental insurance coverage that covers a portion of the co-insurance.

## Footnotes

2 Both drugs are temporarily being covered; as of early 2013, the Minister of Health overruled CVZ's decision.
